# Biosecurity and animal welfare in broiler farms in Mezam division, Cameroon

**DOI:** 10.1016/j.psj.2026.106967

**Published:** 2026-04-16

**Authors:** Asanya Deljose Browstone, Ronald Vougat Ngom, Carine Népidé Ndobadé, Justin Kouamo

**Affiliations:** a Department of Animal Production, School of Veterinary Medicine and Sciences, University of Ngaoundere, Ngaoundere, Cameroon; b Department of Surgery and Medical Pathology, School of Veterinary Medicine and Sciences, University of Ngaoundere, Ngaoundere, Cameroon

**Keywords:** Chicken, Mortality, Poultry, Production, Welfare

## Abstract

Biosecurity is fundamental for disease prevention, productivity, and animal welfare in poultry production systems. In low- and middle-income countries, particularly in conflict-affected regions, farm-level biosecurity and its relationship with animal welfare practices remain insufficiently documented. This study assessed biosecurity compliance in broiler farms in Mezam division (North West, Cameroon) and examined its association with mortality rates and farmers’ knowledge, attitudes, and practices (KAP) regarding animal welfare. A cross-sectional study was conducted between April and September 2025 in 65 commercial broiler farms selected from the lists provided by the regional services in charge of livestock production. Data were collected using structured questionnaires during on-farm face to face interview and technical observations. Biosecurity measures were evaluated using a binary scoring system, and composite scores were expressed as percentages. Animal welfare KAP scores were similarly computed. Overall, 86.2% of farms had a biosecurity score above or equal to 50% and mean biosecurity score of all the surveyed farms was 76.1% (95% CI: 70.5-81.7). The least implemented biosecurity measures were the presence of wheel dips for disinfection, the practice of all-in all-out system, and the removal of carcasses at least twice daily. They were applied by only 35.4%, 35.4% and 47.7% of the farmers, respectively. The average mortality rate per farm was 1.9% by production cycle. Farmers showed a poor knowledge (median score: 38%; 95% CI: 38.1-49.1), negative attitude (median score: 60%; 95% CI: 59.0-67.2) and adequate practices (median score: 70%; 95% CI: 58.9-68.8) toward animal welfare. A moderate positive correlation was recorded between biosecurity implementation and farmers’ practices (r = 0.5, p < 0.001) or knowledge (r = 0.5, p < 0.001) about animal welfare. In addition, mortality rate decreased was associated with biosecurity improvement (r = - 0.3; p = 0.020) in farms. The findings emphasize the need for integrated approaches that combine biosecurity and animal welfare education to enhance poultry production sustainability in resource-limited settings.

## Introduction

Poultry production is one of the fastest-growing livestock subsectors worldwide and plays a crucial role in ensuring food security, income generation, and employment, particularly in low- and middle-income countries (World Bank, 2020; OECD/FAO, 2025). In Cameroon, the poultry sector represents an important component of the agricultural economy, with broiler farming widely practiced in both urban and peri‑urban areas (MINEPIA, 2021). Despite its economic potential, broiler production in the country continues to face substantial challenges, including infectious disease outbreaks, high input costs, limited access to veterinary services, and suboptimal farm management practices (Mouiche et al., 2023; Mpouam et al., 2024; Vougat Ngom et al., 2025). Among these challenges, inadequate biosecurity remains one of the most critical constraints affecting flock health, productivity, and profitability (Ziebe et al., 2025). Biosecurity encompasses a set of preventive measures designed to reduce the risk of introduction, establishment, and spread of infectious agents within and between poultry farms (OIE, 2021). Effective biosecurity has been shown to reduce disease incidence, minimize the need for antimicrobial use, and lower mortality rates in broiler production systems (Racicot et al., 2012; Souillard et al., 2024; Ziebe et al., 2025; Vougat Ngom et al., 2024). Consequently, biosecurity is increasingly recognized as a cornerstone of animal health and economically sustainable poultry production (Mehmedi et al., 2025).

Closely linked to biosecurity is the concept of animal welfare, which refers to the physical and mental state of animals in relation to the conditions in which they live and are managed (Pandolfi et al., 2018; OIE, 2021). In broiler production, animal welfare is influenced by factors such as housing conditions, nutrition, and human-animal interaction (Estevez, 2007; Dawkins et al., 2013). The link between welfare and productivity is widely acknowledged (Henningsen et al., 2018; Pandolfi et al., 2018). In recent years, animal welfare has gained growing attention due to its ethical implications and its relevance to food safety, public health, consumer acceptance and livestock sustainability (Alonso et al., 2020; Papageorgiou et al., 2023). But, to the best of our knowledge, few studies on animal welfare have been performed in Africa where husbandry environment and strategies are different.

The implementation of both biosecurity and animal welfare practices at farm level is strongly influenced by farmers’ knowledge, and attitudes (FAO, 2011). Farmers with adequate knowledge and positive attitudes toward disease prevention and animal welfare are more likely to adopt and maintain effective management practices (Ribbens et al., 2008; Tilli et al., 2023). However, in many developing country settings, formal training opportunities are limited, and farmers often rely on experiential learning or informal advice (Conan et al., 2012; Grace et al., 2015). Socioeconomic factors, production systems, and contextual challenges further shape farm-level decision-making (Gelaude et al., 2014; Bett et al., 2019).

In the **North West (NW)** Region of Cameroon, broiler production occurs within a unique and challenging context. The region has been affected by sociopolitical instability for almost ten years, resulting in disruptions to agricultural activities (Ngwa et al., 2020), reduced access to veterinary and extension services, and constraints on the movement of people and goods. These conditions may compromise the implementation of standard biosecurity measures and animal welfare practices, yet empirical data documenting their status and outcomes remain scarce. Understanding how livestock farmers in this setting manage biosecurity and animal welfare, and how these practices relate to flock mortality, is essential for designing targeted interventions and policy responses (FAO, 2013; World Bank, 2020).

Therefore, the objective of this study was to assess the level of biosecurity implementation in broiler farms of the Mezam division in the NW Region of Cameroon and to examine its relationship with mortality rates and farmers’ **knowledge, attitudes, and practices (KAP)** regarding animal welfare.

## Materials and methods

### Study area

This study was conducted in Mezam division in the NW Region of Cameroon, recognized as one of the major poultry-producing zones in the country (NIS, 2024). With an estimated population of 2 500 000 inhabitants in 2025, the NW Region is characterized by a humid tropical climate with two distinct seasons: a long rainy season extending from March to October and a shorter dry season from November to February. Mean annual rainfall ranges between approximately 1,800 and 2,500 mm, and average temperatures vary between 17.0 and 34.0°C (NIS, 2024).

Mezam division comprises both urban and peri‑urban settings, with a high concentration of small- to medium-scale poultry enterprises supplying broiler meat to local markets. Poultry production in the area is predominantly semi-intensive, with birds housed in deep-litter systems and sourced mainly from commercial hatcheries. These contextual factors make Mezam division an appropriate setting for evaluating biosecurity implementation and animal welfare practices under constrained production conditions.

### Study design

**Fig. 1:**
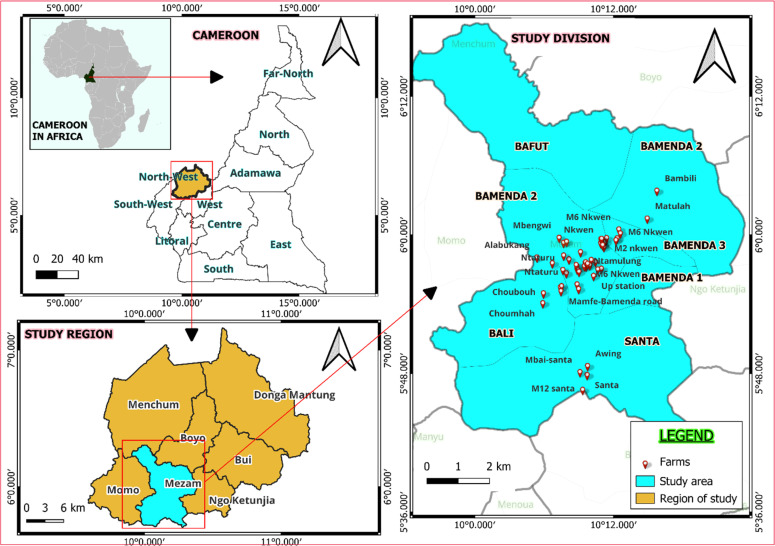
Map of broiler farms studied in the North West Region of Cameroon.

A cross-sectional study was carried out between April and September 2025, covering both the early and peak phases of the rainy season, during which disease challenges in poultry production are typically heightened. The study population consisted of commercial broiler farms operating within Mezam division at the time of the study. Based on a previous study (Vougat Ngom et al., 2025) where biosecurity level in Cameroonian broiler farms was 51%, a minimum sample size of 384 was estimated with the formula of Thrusfield (2007), a precision of 5% and confidence interval of 95%. Only farms with at least 200 broilers and at least two years of operational experience were included to ensure data consistency and relevance. Indeed, these farms are more likely to have stable production systems and established biosecurity practices. Due to the ongoing sociopolitical instability and recurrent attack in the study area, a total of 65 broiler farms who provide their verbal consent were finally enrolled in the study. They were all, selected from the lists of broiler farms obtained at the Regional Delegation of the Ministry of Livestock of the North West localization of all the study farms can be seen in Fig. 1.

### Data collection

Data was collected using a structured questionnaire designed specifically for the objectives of the study. The questionnaire was developed based on existing literature on poultry biosecurity (Tilli et al., 2023) and animal welfare assessments (Alemayehu et al., 2022), as well as practical considerations relevant to broiler production systems in Cameroon. The questionnaire comprised four main sections: (1) socio-demographic characteristics of farmers; (2) biosecurity practices; (3) broiler mortality records; and (4) knowledge, attitudes, and practices regarding animal welfare. The questionnaire was initially drafted in English and subsequently reviewed. Prior to the main survey, the questionnaire was pretested on a small number of broiler farms in Bamenda. Feedback from the pretest was used to refine question wording, improve flow, and minimize ambiguity. The results of this pretest were not included in this study.

The validated questionnaire was used during face-to-face interviews with farm owners or primary farm managers during the farm visits. Interviews done using hard-copy paper forms of the questionnaire were complemented by direct on-farm observations to verify reported practices and to assess the physical condition of housing, equipment, and birds. All interviews and observations were conducted by the same trained personnel with a background in veterinary sciences.

### Assessment of biosecurity practices

Biosecurity practices were categorized into external and internal biosecurity measures, following commonly applied frameworks in poultry biosecurity research (Mallioris et al., 2023; Souillard et al., 2024). External biosecurity measures included practices aimed at preventing the introduction of pathogens into the farm, such as control of visitor access, sourcing of day-old chicks, use of footbaths at farm entrances, and vehicle disinfection. Internal biosecurity measures included practices designed to limit pathogen spread within the farm, such as hand hygiene, use of dedicated equipment, litter management, carcass disposal, cleaning and disinfection routines, and separation of age groups.

Each biosecurity practice was assessed using a binary scoring system, where a score of 1 was assigned if the biosecurity measure was implemented and 0 if it was not. This method allows for a quick and straightforward overview of compliance with recommended practices. Each farm's total biosecurity score was then calculated as a percentage based on the number of implemented practices relative to the total number of assessed measures. Where available, farm records were reviewed to obtain information on flock size, production cycles, and mortality. In farms lacking formal records, farmers were asked to provide best estimates based on the last production batch.

### Farmers’ knowledge, attitudes, and practices toward animal welfare

Animal welfare knowledge, attitudes, and practices were assessed using a set of predefined indicators tailored to broiler production systems (Hemsworth et al., 2015; Madzingira, 2018; Doyle et al., 2021; Alemayehu et al., 2022). Knowledge indicators evaluated farmers’ understanding of basic welfare concepts, including the importance of adequate space, nutrition, health management, and humane handling. Attitude indicators assessed farmers’ beliefs regarding animal welfare and its relevance to productivity and profitability. Practice indicators evaluated the extent to which welfare-friendly management practices were implemented on farms. Data was gathered through direct interviews and complemented with observations of animal handling, housing conditions, feeding, and health management. Responses were scored based on their conformity with recognized animal welfare standards. Answers of the participants were coded using binary system, with 1 representing sufficient/good knowledge, positive attitude, appropriate practice toward animal welfare, and 0 represented poor/low knowledge, negative attitude, and inappropriate practices. As adapted from Subedi et al. (2023), farmers scoring below 70% were considered to have poor knowledge, negative attitudes and inadequate practices whereas those scoring 70% or above were deemed to exhibit good animal welfare understanding, positive attitude and adequate practices. In addition to the fact that this binary scoring system (categorizing as good or poor) help to achieve simplicity, and easy interpretation, it enables straightforward comparisons, and strengthens the study’s conclusions.

### Statistical analysis

Data collected were entered into Microsoft Excel 2013 and subsequently exported for statistical analysis in SPSS version 20 (Chicago, IL, USA). Descriptive statistics, including frequencies, percentages, means, medians, and standard deviations, with 95% confidence interval (CI) were used to summarize socio-demographic, biosecurity scores, mortality rates, and animal welfare KAP scores. After performing normality test, Wilcoxon and Kruskal-Wallis test were used to compare the averages of two and more than two independent groups, respectively. To examine associations between continuous variables, the Spearman correlation test was used. The strength of correlations was interpreted according to Evans (1996) as follow: very weak (0.00-0.19), weak (0.20-0.39), moderate (0.40-0.59), strong (0.60-0.79), and very strong (0.80-1.00). A significance level of 5% was used for all statistical tests.

## Results

### Socio-demographic characteristics of broiler farmers

The socio-demographic characteristics of the broiler farmers included in the study are summarized in Table 1. The majority of farmers were male (58.5%), aged between 36 and 50 years old (41.5%), with a higher level of education (80.0%). A notable number of participants had about 11 years of experience in poultry farming (63%). Poultry farming constituted the primary source of income for a significant proportion of respondents (56.9%), while others engaged in broiler production as a secondary or supplementary economic activity. For example 13.8% were teachers at the secondary schools.

**Table 1:** Variation of biosecurity according to farmer's profile (n = 65).

Variables	Modalities	Frequency (n)	Percentage (%)	Biosecurity score (%)	P-value
Gender	Male	38	58.5	76.4 ± 22.9^a^	0.931
	Female	27	41.5	75.7 ± 22.7^a^	
Age (years)	20-35	22	33.9	71.9 ± 25.3^a^	0.026
	36-50	27	41.5	72.0 ± 20.9^a^	
	> 50	16	24.6	88.8 ± 17.4^b^	
Education level	Secondary	13	20.0	76.7 ± 26.1^a^	0.580
	University	52	80.0	75.9 ± 22.0^a^	
Poultry farming training	Yes	36	55.4	79.5 ± 21.0^a^	0.316
	No	29	44.6	71.8 ± 24.3^a^	
Aviculture as primary activity	Yes	37	56.9	80.1 ± 20.1^a^	0.243
	No	28	43.1	70.8 ± 25.1^a^	
Years of experience	2-11	41	63.1	70.4 ± 23.7^a^	0.036
	12-21	17	26.1	84.6 ± 19.7^b^	
	22-30	7	10.8	88.8 ± 9.0^b^	

For the same variable, value with a different letter (a, b) are statistically different; n: number of farms.

### Characteristics of poultry farms visited

Table 2 shows the characteristics of the studied farms. The majority of farms visited were large farms (64.6%), having 1-5 poultry houses (83.1%), and being 6 to 10 years old (64.6%). Most farmers produced broilers for direct sales (90.8%) and only 9.2% were under contract with hotels and restaurants. A few more than half (52.3%) did not have health agent that followed their farms. For the rest (47.7%), the majority of the health agent were veterinary doctors (48.4%) followed by veterinary nurses (32.2%). Generally, health agents visited farms twice a month (58.1%). The average mortality rate per farm was 1.9%. Mortality rate was highest during the starter phase (1.1%), and declined progressively during the grower (0.5%) and the finisher phase (0.3%) of the production cycle.

**Table 2:** Variation of biosecurity according to farm characteristics (n = 65).

Variables	Modalities	Number of farms	Percentage (%)	Biosecurity score (%)	P-value
Herd size	200–500 (small)	20	30.8	67.4 ± 25.6^a^	0.244
	501–1,000 (medium)	3	4.6	86.5 ± 12.4^a^	
	>1,000 (large)	42	64.6	79.5 ± 20.8^a^	
Number of poultry houses	1-5	54	83.1	73.6 ± 23.1^a^	0.113
	6-10	7	10.8	88.4 ± 18.6^a^	
	> 10	4	6.1	87.8 ± 14.2^a^	
Age of the building (years)*	1-5	9	13.9	56.2 ± 22.5^a^	0.028
	6-10	42	64.6	78.2 ± 22.2^b^	
	> 10	14	21.5	82.4 ± 17.9^b^	
Farm category / Reason for production	Under contract	6	9.2	98.2 ± 3.3^b^	0.004
	Independent	59	90.8	73.8 ± 22.6^a^	
Health agent following the farm	Yes	31	47.7	80.0 ± 21.5^a^	0.443
	No	34	52.3	72.5 ± 23.4^a^	
Qualification of health agent	Breeding technician	6	19.4	81.1 ± 19.8^a^	0.240
	Veterinary doctor	15	48.4	73.3 ± 24.4^a^	
	Veterinary nurse	10	32.2	89.5 ± 14.7^a^	
Frequency of health agent visits	Once per month	3	9.7	80.2 ± 29.6^a^	0.877
	Twice per month	18	58.1	81.2 ± 22.3^a^	
	In case of health problem	10	32.2	77.8 ± 19.8^a^	

For the same variable, value with a different letter (a, b) are statistically different; n: number of farms; * age of the first building was considered if a farm had more than one house.

### Biosecurity level in broiler farms

**Fig. 2:**
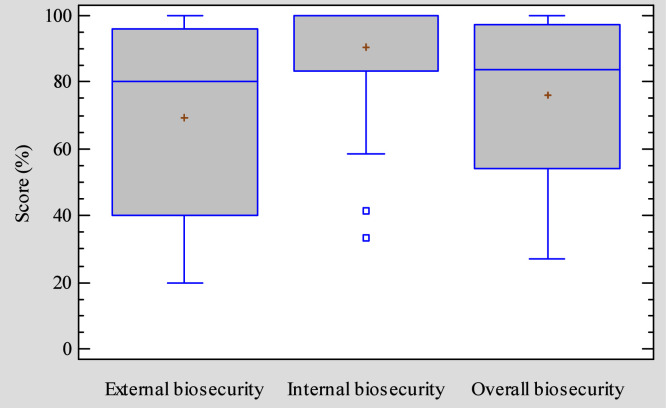
External, internal and overall biosecurity scores amongst the studied farms in the Mezam division (North West, Cameroon).

Overall, 86.2% of the farms achieved biosecurity scores equal or greater than 50%. Figure 2 presents a boxplot comparison of external, internal and overall biosecurity scores of broiler farms. The overall mean score was 76.1% (95% CI: 70.5-81.7). The average scores of external and internal biosecurity were 69.2% (95% CI: 62.3-76.1) and 90.4% (95% CI: 86.7-94.1), respectively. Despite the relatively high overall scores, considerable variability was observed between farms. Some farms demonstrated near-complete implementation of assessed biosecurity measures, while others exhibited notable deficiencies in specific practices.

### Frequency of implementation of biosecurity measures in broiler farms

Table 3 indicates the percentage of farmers that implemented external biosecurity measures. Amongst the 21 external biosecurity measures investigated, five were highly implemented (always by at least 75% of farmers) in broiler farms in the study area. They were: manure storage in isolated specific area (96.9%), rodent control (93.9%), separation of other animals (cattle, pigs etc.) from broiler (92.3%), bird control (86.2%) and specific shoes before entering the poultry house (76.9%). Measures related to no backyard on the site (64.6%), register of visitors (56.9%), delimitation with barrier (52.3%) and washing of hands before entering the poultry house (52.3%) were moderately implemented (always applied by 50 to 75% of farmers).

**Table 3:** Frequency of the implementation of external biosecurity measures on broiler farms in the study area (n = 65).

	Frequency of implementation (%)		
Biosecurity measure	Always	Sometimes	Never
***Animal production, structure and circulation on the site***			
"all-in all-out" poultry production on the site	35.4	27.7	36.9
No backyard on the site	64.6	3.1	32.3
If other animal productions on the site (cattle, pigs) sanitary barriers with poultry (personal, material …)	92.3	0	7.7
Delimitation with a barrier or closure of a professional secured area with only necessary vehicles to the poultry house (feed, chicks, poultry or eggs transport vehicles)	35.4	1.5	46.2
Wheel dips for disinfection of the vehicles or pulverization before entering on the site	35.4	1.5	63.1
***Personnel, visitors or teams***			
Specific clothes before entering the house?	66.2	3.1	30.8
Specific shoes before entering the house?	76.9	0	23.1
washing of hands before entering the house	66.2	12.3	21.5
Showering before entering the house	53.9	6.2	40.0
Specific clothes before entering in the house	52.3	1.5	46.2
Specific shoes before entering in the house	56.9	0	43.1
Washing of the hands before entering in the house	52.3	10.8	36.9
Showering before entering in the house	41.5	6.2	52.3
Register for visitors and teams	56.9	3.1	40.0
***Poultry at arrival***			
Register for the flock (origin, number of poultry, …)	98.5	0	1.5
If the chicks deliverer enters in the house: specific clothes and shoes	49.2	7.7	43.1
***Feed and drinking water of the poultry***			
Feed storage protection	100	0	0
Drinking water analysis end line each year	63.1	1.5	35.4
***Biological vectors***			
Rodents control (deratting or other measures)	93.9	0	6.1
Wild birds control (protection of the ventilation circuit or other measures)	86.2	0	13.8
No domestic animals on the site (pets, dogs or cats),	66.2	1.5	32.3
***Management of manure and management of death animals***			
Manure stored in a specific isolated area outside of the secured professional area (or if no secured area: away from the house)	96.9	0	3.1
Removal of the carcasses at least twice a day	47.7	30.0	21.5
Rendering tank located outside of the secured area (or if no secured area: away from the house) allowing the passage of the truck away from the house	58.5	1.5	40.0
Cleaning and disinfection of the rendering tank after each collection	63.1	0	36.9

**Fig. 3:**
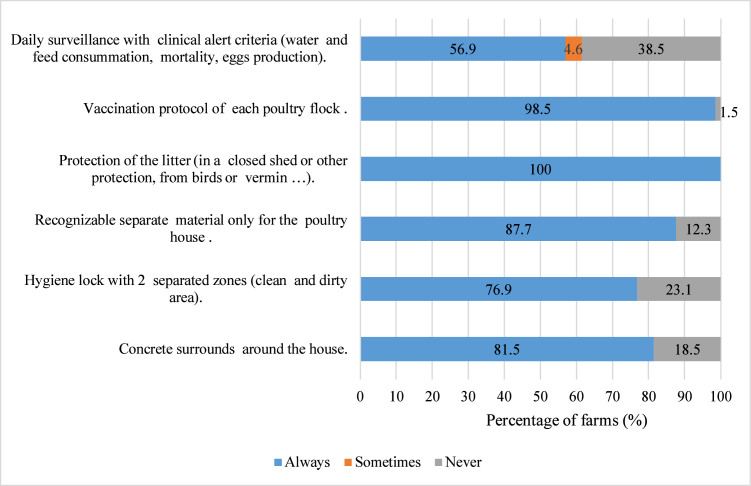
Percentage of level of implementation of biosecurity measures related to structure and circulation in the poultry house, management of the materials and management of poultry (n = 65).

**Fig. 4:**
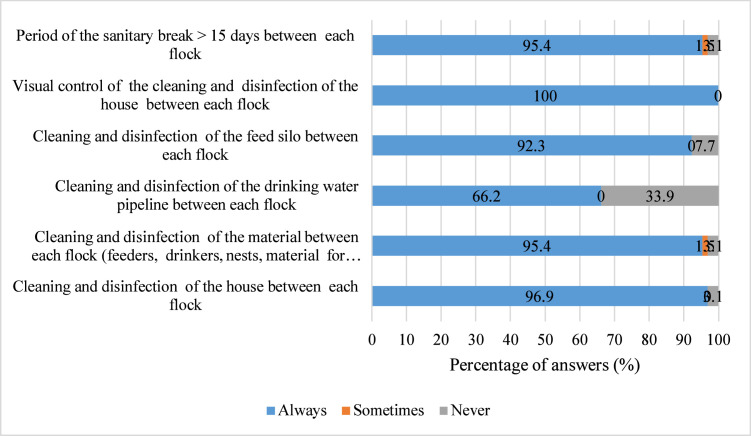
Percentage of level of implementation of biosecurity measures related to cleaning and disinfection of the materials and house (n = 65).

Internal biosecurity measures such as protection of litter (100%), vaccination protocol of each poultry flock (98.5%), period of sanitary break > 15 days (95.4%), cleaning and disinfectant of materials between each flock (95.4%) and recognizable separate materials only for poultry (87.5%) were the most implemented measures (Fig. 3, Fig. 4). Measures related to cleaning and disinfectant of drinking water pipeline between each flock (66.2%) and daily surveillance (56.9%) were moderately implemented.

In general, the biosecurity measures always applied by the minority of broiler farmers were the removal of carcasses at least twice daily (47.7%), the practice of all-in all-out system (35.4%) and the presence of wheel dips for disinfection (35.4%).

### Factors associated with biosecurity compliance

Table 1, Table 2 reveal the variation of biosecurity according to participants’ profile and farm characteristics, respectively. Farmers aged above 50 years had a better biosecurity score (88.8 ± 17.4%) in their farms compared to others. In addition, farmers with 22 to 30 years of experience had higher biosecurity score (88.8 ± 9.0%). Farms which produced under contract recorded a significantly (p = 0.004) high biosecurity score (98.2 ± 3.3%) compared to others (73.8 ± 22.6). In general, no significant variation of the mortality rate was recorded according to biosecurity level although there was a trend towards reduced mortality in farms with good biosecurity. The mortality rate per farm was 3.2 ± 2.2% in the farms with a biosecurity below 50% while farms with a biosecurity score above or equal to 50% had a mortality rate of 1.7 ± 0.9%. Farmers’ gender, or educational level did not influenced biosecurity implementation. Same goes for the number of poultry houses in the farm, presence of health agent and frequency of the visit of health agent in the farm which showed no significant association with biosecurity compliance on broiler farms in the Mezam division in Cameroon.

### Reasons for not implementing biosecurity measures in farms

**Fig. 5:**
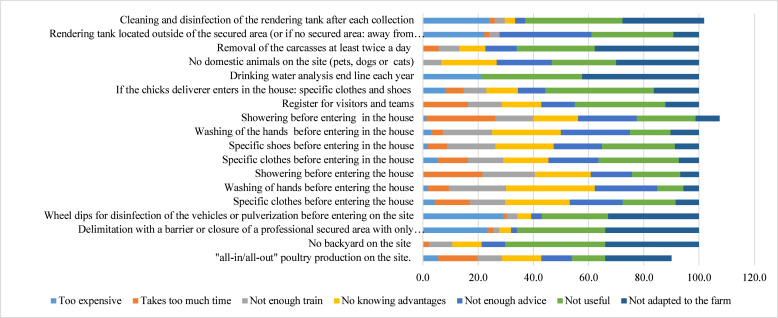
Percentage of reasons for not implementing external biosecurity measures*.* Only biosecurity measures not always implemented by a least 75% of farmers were considered.

**Fig. 6:**
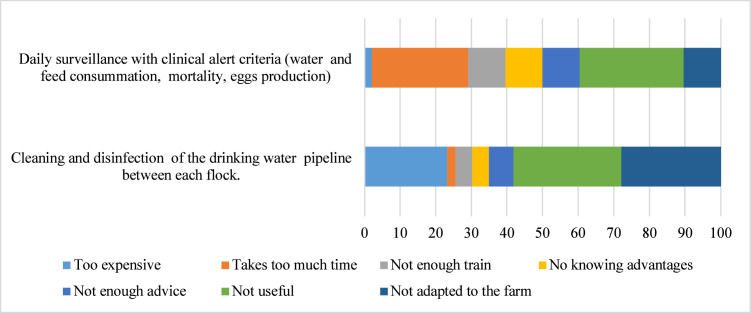
Percentage of reasons for not implementing internal biosecurity measures. Only biosecurity measures not always implemented by a least 75% of farmers were considered.

For the biosecurity measures not always implemented by at least 75% of farms, the reasons for their non-compliance were assessed. The main reasons provided by farmers for not implementing external biosecurity were related to “not useful”, “not adapted to the farm” and “not enough advice" (Fig. 5). For the internal biosecurity measures, the reasons given by farmers were “not useful”, “take too much time” and “not adapted to the farm” (Fig. 6).

### Farmers’ knowledge, attitude, and practice levels regarding poultry welfare

**Fig. 7:**
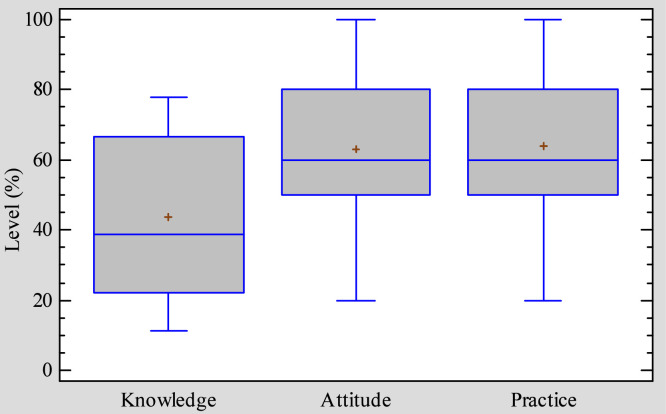
Comparison of knowledge, attitude and practice scores of famers on broiler welfare in the Mezam division (n = 65).

The distribution of farmers' knowledge, attitude and practice scores regarding animal welfare is represented by Fig. 7. Farmers recorded higher scores for attitude and practices with respective median of 60% (95% CI: 58.9-67.2) and 70% (95% CI: 58.9-68.8). Their knowledge of animal welfare was lower with a median score of 38% (95% CI: 38.1-49.1). Based on their profile, only farmers with more than 11 years of experience in poultry farming, those with aviculture as main activity (72.2 ± 17.0%) or with poultry farming training (71.1 ± 17.5%) showed adequate practices concerning animal welfare (Table S1 supplementary material).

Gender, age, and level of education did not influenced knowledge (Table S1 supplementary material), attitude (Table S2 supplementary material) and practices (Table S3 supplementary material) levels of farmers concerning animal welfare while training in poultry farming, having poultry farming as the main activity, and greater years of experience were all linked to significantly higher knowledge and practice scores. Indeed, compared with the corresponding categories of participants, those with poultry farming as main income activity had significantly higher practice (72.2 ± 17.0%) and knowledge (49.5 ± 22.8%) scores on animal welfare. Details concerning the variation of the knowledge, attitude and practice scores of broiler farmers toward animal welfare can be found in supplementary material.

### Relation between biosecurity, mortality and farmers’ KAP toward animal welfare

**Fig. 8:**
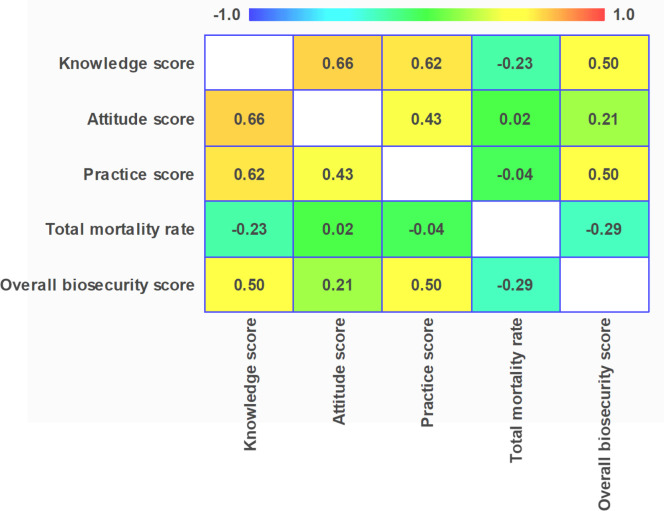
Plot presenting the correlation coefficient between biosecurity, mortality rate and farmers’ knowledge, attitude and practices concerning animal welfare in the Mezam division, Cameroon (n = 65). The strength of the correlations was interpreted as follows: very weak (0.00–0.19), weak (0.20–0.39), moderate (0.40–0.59), strong (0.60–0.79), and very strong (0.80–1.00).

A strong correlation was recorded between farmers’ knowledge and attitude scores (r = 0.7; p < 0.001) and between the knowledge and practice score (r = 0.6; p < 0.001). The correlation between practice and biosecurity implementation was moderate (r = 0.5; p < 0.001). As can be seen in Fig. 8, another moderate correlation (r = 0.5; p < 0.001) was recorded between the level of biosecurity and broiler farmers’ knowledge about animal welfare. Similarly, attitude and practices of farmers concerning animal welfare were moderately associated (r = 0.4; p < 0.001). Results showed that in broiler farms of the study area, mortality rate significantly decreased with biosecurity improvement (p = 0.020). But this was found to be a weak association (r = −0.3).

## Discussion

The present study provides a comprehensive assessment of biosecurity implementation, and farmers’ knowledge, attitudes, and practices regarding animal welfare in a conflict-affected poultry production setting in the NW Region of Cameroon.

The results revealed that the majority of broiler farms (86.2%) achieved a biosecurity score ≥ 50%. Although the ongoing crisis in the NW Region could have been expected to negatively impact biosecurity implementation due to economic constraints and reduced veterinary oversight, the findings suggest otherwise. This paradox may be explained by increased farmer’s awareness of the critical role of biosecurity in safeguarding flocks when veterinary access is limited. When compared with findings by Vougat Ngom et al. (2025) in other regions of Cameroon, our results showed a higher percentage of farms with overall biosecurity score above or equal to 50%. In their study, 59% of surveyed farms had a biosecurity score ≥ 50%. The difference observed may be associated with a greater proportion of farmers in the Mezam division who had poultry farming as main income activity, had received poultry farming training, and had more years of farming experience, with the latter factor shown in our analysis to be positively associated with biosecurity compliance. In addition, the biosecurity assessment methods used played a role. In this study, we employed a binary scoring system in which each measure was recorded as either applied or not, a method that can result in a higher proportion of farms being classified as having biosecurity score ≥ 50% meanwhile in the study of Vougat Ngom et al. (2025), Biocheck.UGent® tool was used, which applies a weighted and scoring approach. This difference in methodology likely explain the variation in the overall scores reported between the two studies. The difference in the methods used can also justify the divergence between the findings of this study with the low biosecurity level reported by Mahmood et al. (2025) in broiler farms in Paskistan. Comparatively, study by Adomako et al. (2024) in the Ashanti Region of Ghana reported that only 62% of broiler farms met the threshold for a biosecurity score above 50%.

The average overall biosecurity score recorded in this study was relatively high (76.1%). This encouraging level may be explained by several factors: (i) the training in poultry farming received by the majority of farmers which likely improved their knowledge and consistent application of biosecurity measures, (ii) the fact that in addition to having poultry farming as their main activity, the majority of farmers in the study had several years of experience in poultry production, which can strengthen awareness of disease risks and the economic impact of preventive practices, (iii) the significant number of farms reported being regularly followed by a health agent, who could provide technical guidance and reinforce compliance with biosecurity protocols. The average biosecurity score in this study is higher than that reported by Chowdhury et al. (2023) in Bangladesh (58.3%), Mdemu et al. (2025) in Tanzania (58.2%), and Dhakal et al. (2025) in Nepal (33.4%), where generally the proportion of poultry farmers receiving specialized training or regular veterinary follow-up was comparatively lower. The stronger implementation of internal biosecurity measures compared with external measures observed in this study aligns with findings from previous research in poultry systems (Tanquilut et al., 2020; Mirzaie et al., 2023; Alam et al., 2025; Vougat Ngom et al., 2025). This might indicate that farmers perceived internal biosecurity measures as the main drivers to reduce disease occurrence on farms.

The least implemented biosecurity measures in this study were the presence of wheel dips for disinfection (35.4%), all-in all-out practice (35.4%), and the removal of carcasses at least twice daily (47.7%). This finding can be explained by practical and economic constraints faced by farmers. The lack of implementation of these biosecurity measures can have significant negative implications for flock health and overall farm productivity. Installing and maintaining wheel dips requires additional infrastructure and regular replenishment of disinfectant solution, which can be perceived as costly or inconvenient. The absence of wheel dips facilitates the mechanical transmission of pathogens through vehicle movement, increasing the risk of introducing infectious agents onto farms. A study by Aguidissou et al. (2020) reported that 8% of poultry farms surveyed had a wheel dips in Benin. Similarly, applying an all-in all-out production system often demands higher levels of coordination and investment, which may not be feasible for farmers who rely on continuous production to sustain income. Failure to implement an all-in all-out practices allows continuous circulation of pathogens between flocks, making disease elimination difficult and increasing the persistence of infections within the farm environment. In Bangladesh, Akter et al. (2025) found that 93.3% of broiler farms adhered to an all-in all-out system. The difference with the current study could be related to farm characteristics and farmer demographics such as herd size and years of experience in chicken farming. Regular carcass removal, while critical for disease control, also requires sufficient labor and strict daily routines which are often difficult to maintain in resource-limited production systems. Inadequate carcass removal can further exacerbate disease spread, as decomposing carcasses serve as reservoirs for pathogens and attract scavengers or insects that can act as vectors (Grace et al., 2024; Souillard et al., 2025). Similarly, in Pakistan, carcass removal was among the weakest biosecurity practices on broiler farms (Mahmood et al., 2025). Carcass removal was among the least implemented biosecurity measures in Kenya (Kemunto et al., 2025). Improving this practice is necessary to reduce disease transmission.

This study revealed several reasons for non-compliance of the above mentioned biosecurity measures within the Mezam division. The interviewed stakeholders commonly defined the unimplemented biosecurity as “not useful” or “not adapted” to the broiler farms. This highlights the need for a well-tailored biosecurity training where farmers can learn about the importance of measures such as presence of wheel dips for disinfection, all-in all-out practice, and the carcasses management in preventing and controlling infectious diseases. Besides other reasons not listed, similar responses were perceived by Italian poultry farmers as the main reasons for not implementing on-farm biosecurity measures (Laconi et al., 2023).

The findings of this study showed a poor knowledge level (median score of 38%) of farmers regarding animal welfare. This insufficient knowledge could be attributed to the fact that animal welfare is still a developing concept in Cameroon and many other African countries, and most training programs traditionally focus more on production, disease prevention, and profitability rather than on welfare-specific standards and principles. Moreover, the absence of clear national policies or guidelines explicitly addressing animal welfare have contributed to the lack of structured learning opportunities for farmers. Despite this, a minority of farmers especially those with poultry farming as main income activity or those with more years of experience achieved relatively better knowledge, showing that individual background plays a role. Contrary to our observations, Wahyuwardani et al. (2025), found a better farmers’ knowledge level about animal welfare in Indonesia. The difference observed could be attributed to the better integration of animal welfare topics into livestock production chain in this country (Sinclair et al., 2019), reflecting national efforts to align with international standards.

Data analysis showed that farmers had a median attitude score of 60% toward animal welfare. This result may be linked to farmers’ practical experience in poultry production: even though animal welfare is still a new concept and not explicitly emphasized in training programs, many farmers recognize from experience that poor welfare conditions (such as overcrowding, poor housing, or inadequate handling) negatively affect flock health and productivity. Thus, while formal knowledge of welfare standards remains limited, farmers’ everyday observations of the link between management practices and production likely contributed to their relatively positive attitudes. Additionally, cultural and ethical considerations may lead farmers to value animal welfare, even without detailed technical knowledge. The livestock farmers’ attitude level in this study is almost similar than that reported by Wahyuwardani et al. (2025) in Indonesia (59.8%). In opposite, attitude scores of farmers in the Mezam Division were below those found by Alemayehu et al. (2022) in Ethiopia (3.4 out of 5). The difference may reflect the need of more sensibilization on the topic in Cameroon.

Adequate farmers’ practices toward animal welfare was detected in this study, with a median score of 70%. In contrast, lower practice scores were reported by Alemayehu et al. (2022) in Ethiopia. This suggests that, despite relatively low formal knowledge of animal welfare as a concept, farmers still apply a variety of welfare-related practices as part of their routine flock management. This may be because many welfare-friendly practices, such as maintaining clean litter, avoiding overcrowding, and providing adequate feed and water, overlap with traditional training on disease prevention and productivity. Farmers may therefore adopt these practices primarily for their visible benefits in reducing mortality and improving growth performance, rather than from an explicit understanding of animal welfare principles.

Findings from the current study showed a moderate association between farmers’ knowledge (r = 0.5; p < 0.001) and practices (r = 0.5; p < 0.001) about animal welfare and biosecurity implementation. This could be explained by the link between animal welfare and biosecurity, which both aim to improve animal health (Dewulf and van Immersel, 2019). Biosecurity measures help to maintain poultry welfare. Improving animal welfare sometime requires changes to infrastructure and other biosecurity measures. Indeed, on-farm practices to assure animal welfare and biosecurity measures are sometimes similar. For example, adequate housing or water of good quality are part of biosecurity that is also helpful to improve animal welfare. In addition, stringent on farm disinfection can mitigate disease transmission, thereby improving poultry health and welfare. The link between welfare and biosecurity in poultry farms has been previously reported (Kovacs et al., 2025). The strong relationship between farmer’s knowledge and attitude toward animal welfare, indicating that greater knowledge is associated with more favorable attitude was also reported by Wahyuwardani et al. (2025).

### Study limitation

This study has certain limitations that should be considered when interpreting the findings. The low number of farms involved in the study may limit the generalizability of the results to all broiler farms in the study region. In addition, reliance on self-reported data for some variables such as mortality may introduce reporting bias, although on-farm observations were used to mitigate this limitation. Furthermore, the interpretation of significant differences observed for some variables with uneven distribution (farm category/reason for production and years of experience) should be made with caution. Despite these constraints, the study provides valuable insights into biosecurity and welfare practices in an under-researched setting.

### Conclusion

This study revealed that the level of biosecurity implementation in broiler farms within the North West region of Cameroon was generally good. However, a few measures such as the use of wheel dips, strict all-in all-out production systems, and prompt carcass disposal were less applied. These gaps suggest that although the majority of farmers maintain satisfactory biosecurity standards, there is still room for improvement. The reasons for not implementing biosecurity measures were largely linked to the fact that farmers perceived certain practices as unnecessary or lacked awareness of the consequences of neglecting such measures. This suggests that continuous sensitization that emphasize the long-term benefits of preventive health management, infrastructural support, and monitoring could help sustain poultry production in the study area. The disparity between insufficient animal welfare knowledge, negative attitude and adequate practice observed among broiler farmers is a noteworthy finding. While most farmers implemented basic welfare-friendly practices, their limited formal knowledge suggests that these practices may be driven by experiential learning rather than a conceptual understanding of welfare principles. This disconnect has important implications for long-term sustainability. Without adequate knowledge, farmers may be less equipped to identify subtle welfare problems, adapt practices to changing conditions, or comply with emerging welfare standards. The findings highlight the need for targeted educational interventions that go beyond promoting good knowledge and focus on building a solid understanding of animal welfare concepts and indicators.

### Implications for poultry production in conflict-affected settings

The study context adds an important dimension to the interpretation of the findings. In regions affected by sociopolitical instability, access to veterinary services, extension support, and production inputs is often constrained. Despite these challenges, the relatively high biosecurity compliance observed suggests resilience and adaptability among broiler farmers. However, persistent gaps in external biosecurity and welfare knowledge underscore the need for context-specific interventions that account for security, mobility, and resource limitations. Integrating biosecurity and animal welfare training into existing farmer networks, cooperatives, and contractual arrangements may offer a practical approach to improving management practices under such conditions. Furthermore, strengthening surveillance and record-keeping systems could enhance farmers’ ability to monitor mortality and welfare outcomes more effectively.

## Funding

This research received no external funding.

## Ethics approval and consent to participate

This study was approved by the scientific research and ethics committee of the School of Veterinary Medicine and Sciences of the University of Ngaoundere (2025/020UN/ESMV/DAARCS/SSFC of 27 March 2024) and the Ministry of livestock of Cameroon (MINEPIA/DREPIA/NW/104/40). Informed consent was obtained from all study participants.

## Data availability statement

The raw data supporting the conclusions of this article will be made available by the corresponding author on request.

## CRediT authorship contribution statement

**Asanya Deljose Browstone:** Conceptualization, Methodology, Formal analysis, Investigation, Data curation, Writing – original draft, Writing – review & editing, Visualization. **Ronald Vougat Ngom:** Writing – review & editing, Writing – original draft, Validation, Supervision, Methodology, Formal analysis, Conceptualization, Data curation, Visualization. **Carine Népidé Ndobadé:** Methodology, Validation, Writing – review & editing, Supervision. **Justin Kouamo:** Conceptualization, Validation, Writing – review & editing, Supervision.

## Disclosures

The author(s) declare(s) that there is no conflict of interest regarding the publication of this paper.

## Appendix Supplementary materials

Image, application 1

## Footnotes

Supplementary material associated with this article can be found, in the online version, at doi:10.1016/j.psj.2026.106967.
