# Supplementary material for: Biosecurity and animal welfare in broiler farms in Mezam division, Cameroon
Source: Poult Sci. 2026 Apr 16;105(8):106967. doi: 10.1016/j.psj.2026.106967 (PMC13199986; doi:10.1016/j.psj.2026.106967)
Supplement: Supplementary file 1 [file mmc1.docx]

**Biosecurity and Animal Welfare in Broiler farms in Mezam Division,** **Cameroon**

**Supplementary materials**

Table S1: Knowledge scores of broiler farmers on animal welfare according to their profiles

| Variables | Modalities | Mean score ± SD (%) | P-value |
| --- | --- | --- | --- |
| Gender |  |  |  |
|  | Male (n = 38) | 47.2 ± 22.6^a^ | 0.104 |
|  | Female (n = 27) | 38.5 ± 21.1^a^ |  |
| Age (years) |  |  |  |
|  | 20-35 (n = 22) | 37.6 ± 22.1^a^ | 0.156 |
|  | 36-50 (n = 27) | 43.6 ± 21.7^a^ |  |
|  | > 50 (n = 16) | 51.7 ± 22.2^a^ |  |
| Educational level |  |  |  |
|  | Secondary (n = 13) | 46.6 ± 19.6^a^ | 0.557 |
|  | University (n = 52) | 42.8 ± 23.0^a^ |  |
| Poultry farming training |  |  |  |
|  | Yes (n = 36) | 49.5 ± 22.8^b^ | 0.029 |
|  | No (n = 29) | 36.2 ± 19.5^a^ |  |
| Aviculture as primary activities |  |  |  |
|  | Yes (n = 37) | 50.1 ± 22.7^b^ | 0.010 |
|  | No (n = 28) | 34.9 ± 18.7^a^ |  |
| Years of experience in poultry farming |  |  |  |
|  | 2- 11 (n = 41) | 36.9 ± 20.6^a^ | 0.007 |
|  | 12- 21 (n = 17) | 52.6 ± 22.7^b^ |  |
|  | 22- 30 (n = 7) | 61.1 ± 13.6^b^ |  |

For the same variable, values with different letters of the alphabet (a, b, c) are significantly different at *p* > 0.05; SD: standard deviation; n: sample size; n: number of farms.

Table S2: Attitude scores of broiler farmers on animal welfare according to their profiles

| Variables | Modalities | Mean score ± SD (%) | P-value |
| --- | --- | --- | --- |
| Gender |  |  |  |
|  | Male (n = 38) | 64.5 ± 17.8^a^ | 0.342 |
|  | Female (n = 27) | 61.1 ± 15.0^a^ |  |
| Age (years) |  |  |  |
|  | 20-35 (n = 22) | 60.0 ± 20.0^a^ | 0.492 |
|  | 36-50 (n = 27) | 65.6 ± 15.8^a^ |  |
|  | > 50 (n = 16) | 63.1 ± 13.0^a^ |  |
| Educational level |  |  |  |
|  | Secondary (n = 13) | 66.1 ± 12.6^a^ | 0.548 |
|  | University (n = 52) | 62.3 ± 17.69^a^ |  |
| Poultry farming training |  |  |  |
|  | Yes (n = 36) | 65.3 ± 19.3^a^ | 0.101 |
|  | No (n = 29) | 60.3 ± 12.4^a^ |  |
| Aviculture as primary activities |  |  |  |
|  | Yes (n = 37) | 66.5 ± 18.3^b^ | 0.003 |
|  | No (n = 28) | 58.6 ± 13.2^a^ |  |
| Years of experience in poultry farming |  |  |  |
|  | 2-11 (n = 41) | 59.8 ± 17.4^a^ | 0.002 |
|  | 12-21 (n = 17) | 70.0 ± 15.8^b^ |  |
|  | 22-30 (n =7) | 65.7 ± 7.98^ab^ |  |

For the same variable, values with different letters of the alphabet (a, b, c) are significantly different at *p* > 0.05; SD: standard deviation; n: sample size; n: number of farms

Table S3: Animal welfare practice scores of broiler farmers according to their socio-demographics

| Variables | Modalities | Mean score ± SD (%) | | P-value |
| --- | --- | --- | --- | --- |
| Gender |  |  | |  |
|  | Male (n = 38) | 63.2 ± 22.7^a^ | | 0.771 |
|  | Female (n = 27) | 64.8± 15.3^a^ | |  |
| Age (years) |  |  | |  |
|  | 20-35 (n = 22) | 56.4 ± 18.4^a^ | | 0.076 |
|  | 36-50 (n = 27) | 66.3 ± 18.6^ab^ | |  |
|  | > 50 (n = 16) | 70.0 ± 21.6^b^ | |  |
| Educational level |  |  | |  |
|  | Secondary (n = 13) | 57.7 ± 20.58^a^ | | 0.167 |
|  | University (n = 52) | 65.4 ± 19.5^a^ |  | |
| Poultry farming training |  |  | |  |
|  | Yes (n = 36) | 71.1 ± 17.5^b^ | | 0.001 |
|  | No (n = 29) | 54.8 ± 19.0^a^ | |  |
| Aviculture as primary activities |  |  | |  |
|  | Yes (n = 37) | 72.2 ± 17.0^a^ | | <0.001 |
|  | No (n = 28) | 52.9 ± 18.0^a^ | |  |
| Years of experience in poultry farming |  |  | |  |
|  | 2-11 (n = 41) | 55.6 ± 17.3^a^ | | <0.001 |
|  | 12-21 (n = 17) | 72.9 ± 16.1^ab^ | |  |
|  | 22-30 (n = 7) | 90.0 ± 0.0^b^ | |  |

For the same variable, values with different letters of the alphabet (a, b, c) are significantly different at *p*> 0.05; SD: standard deviation; n: sample size; n: number of farms.
